# Residue depletion profile and withdrawal interval estimation of ivermectin in eggs following topical administration of injectable ivermectin to domestic chickens (*Gallus domesticus*): a pilot study

**DOI:** 10.3389/fvets.2025.1527808

**Published:** 2025-02-06

**Authors:** Melissa A. Mercer, Jennifer L. Davis, Scott E. Wetzlich, Maaike O. Clapham, Lisa A. Tell

**Affiliations:** ^1^Department of Veterinary Medicine and Epidemiology, School of Veterinary Medicine, University of California, Davis, Davis, CA, United States; ^2^Department of Biomedical Sciences and Pathobiology, Virginia Maryland College of Veterinary Medicine, Blacksburg, VA, United States

**Keywords:** ivermectin, egg withdrawal interval, poultry, pharmacokinetics, drug residues, egg residues

## Abstract

**Introduction:**

Topical ivermectin is commonly prescribed extra-label for the control of mite infestations in backyard chicken flocks in the US.

**Methods:**

Domestic laying hens (*n* = 8; 78 weeks of age, weight 1.7–2.2 kg) were administered injectable ivermectin solution topically over the jugular vein (0.4 mg/kg every 7 days for 2 doses). Ivermectin concentrations in egg white and egg yolk were determined using UPLC with fluorescence detection.

**Results:**

The average period between eggs laid was 1.52 days. Ivermectin preferentially distributed to the egg yolk with an observed *C*_max_ of 3.54 ng/g occurring at observed *T*_max_ of 6.6 days and a *T*_1/2_ of 9.5 days. Residues persisted at low concentrations in egg yolk for up to 71 days after the final dose. WDIs for the egg yolk matrix were estimated using the FDA, EMA, and terminal-elimination half-life multiplier methods (HLM). The longest estimated WDI was 102 days for the EMA 95/95 method (95% confidence interval for 95th population percentile) with the limit of detection (LOD; 0.03 ng/g) set as the maximum residue limit. The FDA 95/99 method using the LOD as the tolerance estimated an 81 day WDI, the HLM method estimated a 96 day WDI.

**Discussion:**

This study improves the understanding of the residue depletion kinetics of ivermectin in eggs after topical administration to older hens with inconsistent egg production. Ivermectin is systemically absorbed following topical administration of the injectable formulation in domestic egg laying chickens, resulting in prolonged egg residues. Ivermectin is preferentially distributed to the egg yolk over the egg white following topical administration of the injectable formulation in egg laying chickens. Since plasma kinetics were not evaluated, the impact of systemic exposure on egg residue kinetics following topical administration remains unknown. The results provide insight into how the estimated ivermectin egg WDIs using regulatory methods differ based on the maximum residue limit/tolerance applied and portion of the terminal elimination phase sampled.

## 1 Introduction

Domestic backyard poultry ownership is popular in the US in both rural and urban settings, where producers keep small flocks to provide meat and/or eggs for personal use or for local food systems. However, despite the sustained popularity of backyard poultry ownership, most backyard poultry owners have poor engagement with veterinary services—citing difficulty in accessing veterinary care, and high cost of treatment (1, 2). Ectoparasites are a common health concern in chickens, and are common in backyard poultry flocks (3, 4). The northern fowl mite (*Ornithonyssus sylviarum)* is the most common ectoparasite affecting commercial poultry in the US, leading to significant animal damage and economic losses in both commercial and backyard poultry (5). Mite infestations in chickens can lead to anemia, transmissible diseases, reduced egg quality, negative impacts on animal welfare, and death in affected chickens (4–7). As welfare concerns for food producing animals are a highly cited motivator for individuals to keep backyard poultry, effective veterinary intervention for mite infestations is key to improving animal welfare and meeting owner expectations (2).

Eradication of northern fowl mite infestation in flocks is incredibly challenging, and resistance to previously effective measures such as topical application of permethrins and pyrethroids is widespread (5). Due to backyard poultry owners viewing their animals first as pets before food producing animals, there is increasing need for individual-animal control measures for mite infestation. While no specific studies have been performed evaluating its efficacy against the northern fowl mite, ivermectin has been shown to be effective against the red fowl mite, leading to its extra-label use in backyard chicken flocks for treatment of the northern fowl mite in the US (6, 8). As the majority of backyard poultry owners do not routinely administer medications to their flocks, the use of topical ivermectin is a widespread and attractive treatment option as it is non-invasive and requires minimal animal stress and handling (2, 9).

Since ivermectin is not approved for use *via* any route of administration in any poultry species in the US, there is no tolerance for egg or meat residues, and any use of ivermectin is considered extra-label drug use (ELDU). In the US, backyard poultry flocks are mostly kept for egg production for human consumption, and relatively few backyard flocks are raised for meat production (2). According to the Animal Medicinal Drug Use Clarification Act of 1994 (AMDUCA) (10) and the regulations outlined in US Code of Federal Regulations 21 CFR 530 (11), licensed veterinarians with a valid veterinary patient-client-relationship are permitted to prescribe FDA-approved drugs in an extralabel manner. Under these same regulations, following any ELDU in food producing animals in the US, a greatly extended withdrawal interval (WDI) for any food products must be issued by the prescribing veterinarian to protect human food safety and ensure that illegal drug residues do not occur (10, 11).

There are several different approaches for estimating ELDU WDIs. The simplest method is by leveraging the assumption that >99% of a drug is depleted from a tissue after 10 elimination half-lives (12). Therefore, the terminal elimination half-life method simply multiplies the terminal tissue or product half-life by a factor of 10 (12). The regulatory approaches, however, rely on statistical methods to establish a withdrawal period for the 95th percentile of population (European Medicines Agency, EMA) or the 99th percentile of the population (US Food and Drug Administration, FDA) with a 95% confidence interval using software applications. Since the regulatory methods base their withdrawal periods on a representative range of a population mean, in comparison to the sample mean of the terminal elimination half-life approach, regulatory methods may have the benefit of offering a more conservative ELDU WDI estimate that considers a wider population variance. However, the regulatory methods assume a homogenous population of animals, and a normally distributed dataset derived from a good laboratory practice study—which is not always feasible in the small studies funded to examine residue depletion following ELDU in food animals.

The US Food Animal Residue Avoidance Databank (FARAD) is a federally funded program that serves to help US veterinarians by providing a free of charge service to calculate scientifically based withdrawal interval recommendations following ELDU in the US (13). According to internal data from US FARAD ivermectin was the 6th most commonly requested drug for withdrawal recommendations in poultry in 2023—highlighting a need for evidence based egg withdrawal recommendations following the use of ivermectin in poultry (14). Additionally, internal data from US FARAD found the most common route and dose for administration of ivermectin to poultry was *via* topical application of ivermectin injectable solution at 0.4 mg/kg every 7 days for 2 applications (14). The elimination of ivermectin residues from eggs has been described following intravenous, subcutaneous, and oral administration routes in chickens, and this data has been used by FARAD to calculate estimated WDIs for ELDU in laying chickens *via* those routes (9, 15, 16). However, to date there have been no studies describing the residue kinetics or calculated withdrawal interval following topical administration of ivermectin in chickens. Therefore, the purpose of this study is to characterize the egg residue elimination kinetics and estimate egg withdrawal intervals for ivermectin in chickens following topical administration of ivermectin injectable solution at 0.4 mg/kg every 7 days for a total of two applications.

## 2 Materials and methods

### 2.1 Animals

Adult commercial egg-laying hens (*n* = 8, Hy-line^®^ W-36, Des Moines, IA, USA) that were 78 weeks of age with body weights ranging from 1.7 to 2.2 kg were used in this pilot study. All laying hens were considered healthy based on physical examination but were considered to be in the later stages of their production cycle and therefore were selected to be more representative of an older backyard flock population. The hens were housed individually within view of other chickens in a climate-controlled room of the University of California, Davis Hopkins Avian Facility in wire cages, with a 16 h light and 8 h dark cycle. A commercial poultry feed (16% Layer Crumble Pak, Bar ALE, Williams, CA, USA), drinking water, oyster shell and ground performance-enhancing supplements (Calf Manna Pro, Chesterfield, MO, USA) were provided ad libitum. All animal procedures were performed in accordance with a protocol (IACUC Protocol Number 23096) approved by the Institutional Animal Care and Use Committee of the University of California at Davis.

### 2.2 Experimental protocol

An FDA approved ivermectin product (VetOne Vetrimec 1% (ivermectin) Injection; ANADA 200-447; Bimeda Animal Health Inc, Cambridge, ON, Canada) was applied topically over the jugular vein at a dose of 0.4 mg/kg every 7 days for two applications to domestic egg laying hens (*n* = 8). The dose administered was based on the most common dose, frequency, and product submitted to US FARAD for estimated WDI following ELDU of topical ivermectin in chickens (14). Topical application was performed by parting the feathers over the jugular vein and applying ivermectin *via* calibrated pipet (Pipetman, Gilson, Middleton, WI). Prior to administration of ivermectin, all chickens were weighed to ensure dose accuracy. Eggs were collected immediately prior to dosing, and then every 24 h for 90 days, or until the hen either (a) ceased laying or (b) had 3 consecutive eggs below the limit of detection for ivermectin in both the egg white and egg yolk. The hens were monitored daily for appetite, attitude, and general appearance. Fully formed hard-shelled eggs were stored refrigerated until analysis. The average time from collection to assay was 33.0 days (range 1–67 days).

### 2.3 Ivermectin analysis

Ivermectin concentrations in egg yolk and egg white were determined based on a previously published high performance liquid chromatography (HPLC) with fluorescence detection method with slight modifications (9). Ivermectin was of analytical grade and a European Pharmacopeia reference standard (Santa Cruz Biotechnology, Dallas, TX). Moxidectin was used as the internal standard (Sigma Aldrich, St. Louis, MO). HPLC-grade methanol, HPLC-grade acetonitrile, N-methylimidazole, and trifluoroacetic anhydride were purchased from Fisher Scientific (Fisher Chemical, Fair Lawn NJ). Purified water was obtained with a Nanopure water system (Barnstead, Dubuque, IA, USA). Egg yolks and whites were manually separated and weighed prior to mixing by hand. Two gram aliquots of egg white and yolk were weighed. The aliquots were spiked with 50 uL of the internal standard (moxidectin), and 3 mL of acetonitrile was added to each aliquot prior to mixing for 10 min on a high-speed vortexing shaker. After which, the samples were sonicated and centrifuged at 1,200 g for 10 min. The supernatant was transferred to a clean tube and sample was re-extracted with an additional 3 ml of acetonitrile. Four milliliter of water was added to the total supernatant and was then transferred to pre-conditioned C18 cartridges (Bond Elut, 500 mg, 6 ml, Agilent Technologies, Santa Clara, CA) for solid phase extraction. The C18 cartridges were conditioned with 3 mL of HPLC grade methanol followed by 3 mL of HPLC grade water. The supernatant was applied, and then washed with 2 mL of 1:4 methanol/water, air dried for 3 min, then eluted with 3 mL of HPLC grade methanol. The extractant was transferred to a new tube and evaporated to dryness at 45°C with a gentle stream of nitrogen, and then reconstituted with 100 uL of a 1:1 N-methylimidazole/acetonitrile solution. Derivatization was initiated with 150 uL of a 1:2 solution of trifluoroacetic anhydride/acetonitrile, and then a 200 uL aliquot of the derivatized sample was transferred to autosampler vials for analysis on the chromatographic system. The UPLC system consisted of an Acquity UPLC system with an Acquity fluorescence detector (Waters Corp, Milford, MA). Separation was achieved on an Acquity UPLC BEH C18, 1.7 um, 2.1 × 50 mm column (Waters Corp, Milford, MA). The column temperature was maintained at 30°C and the samples were kept at 10°C. The mobile phase was 60:40 acetonitrile: methanol set at a flow rate of 0.25 mL/min. Injection volume was 5 μL. The fluorescence detector was set at 365 nm excitation and 475 nm emission and the total run time was 3 min.

### 2.4 Standards and quality control sample preparation

Acetonitrile was used as the solvent to prepare the primary stock solution of ivermectin (100 ug/mL). A series of working standard solutions (1,000–5 ng/mL) was created. Similarly, the stock solution of moxidectin (100 ug/mL) was also prepared under the same protocol as the internal standard solution and a 250 ng/mL internal standard working solution was diluted from the moxidectin stock solution. A standard curve was generated *via* mixture of equal volumes of ivermectin working solution and moxidectin internal standard working solution (0.025–50 ng/mL). Three different concentrations of quality control samples were prepared for egg yolk (0.075, 2.5, and 50 ng/g), and for egg white (0.025, 2.5, 50 ng/g), along with blank Quality Control (QC) samples with and without the internal standard, from the control domestic chickens that did not receive any ivermectin for each analysis.

### 2.5 Method validation

Egg yolk and egg white matrices were validated according to the FDA Bioanalytical Method Validation Guidance for Industry (17). Five replicates at each concentration were calculated on a single day for intra-day precision, five replicates at each concentration were calculated over three consecutive days for inter-day precision. The ratio of ivermectin to the internal standard peak areas with 1/*X*^2^ weighting was created for calibration curves. The limit of detection (LOD) was calculated using blank quality controls for each matrix analyzed with each sample set with three times the standard deviation of baseline measurements added. The lower limit of quantification (LLOQ) was measured as five times the standard deviation of the baseline measurement according to the FDA Guidance for Industry (17).

### 2.6 Ivermectin stability testing

The stability of ivermectin in both egg yolk and egg white was assessed with spiked samples since some study samples were refrigerated for up to 67 days following collection and prior to analysis. Briefly, 3 sets of egg yolks and egg whites from untreated hens were spiked with ivermectin in replicates of 0.075, 2.5, and 50 ng/g for egg yolks and 0.025, 2.5, and 50 ng/g for egg whites. Each sample at each spiked level for each matrix was analyzed at the time of fortification and then stored in a 4.5°C refrigerator. Each sample at each spiked level for each matrix was then re-analyzed at 61 days post-storage.

### 2.7 Pharmacokinetic analysis

Ivermectin concentrations in egg whites were below the LLOQ in the majority of samples, so pharmacokinetic analysis could not be performed in this matrix. Concentration vs. time data for egg yolk residues was used to estimate egg yolk pharmacokinetic parameters using a commercial software program (Phoenix WinNonlin 8.3, Certara, Princeton, NJ, USA) and a non-compartmental analysis approach. The maximum concentration (*C*_max_) in egg yolk and time to maximum concentration (*T*_max_) in egg yolk were observed directly from the data. Terminal elimination-half lives were estimated using the best fit data points. The pharmacokinetic parameters were calculated as follows: area under the egg yolk concentration-time curve extrapolated to infinity (AUC_0−∞)_ using the linear trapezoidal method, elimination rate constant (λ_z_) using a linear regression of the terminal log-linear portion of the egg yolk concentration profile, terminal elimination half-life (*t*_1/2_) using the quotient of dividing the natural log of 2 by the elimination rate constant. Concentration vs. time data for the egg yolk residues were plotted using a commercial graphing software (GraphPad Prism 10.2.1, GraphPad Software, La Jolla, CA, USA).

### 2.8 Estimation of egg withdrawal intervals

The terminal elimination half-life approach was used to estimate a WDI by multiplying the calculated *t*_1/2_ for ivermectin in egg yolk by a factor of 10 to estimate the time when 99% of the drug would be depleted from the tissue. Since ivermectin is not currently approved for use in layers in the US, Canada, or EU, no tolerance or maximum residue limit (MRL) currently exists. Therefore, based on US 21 CFR 530.22(a) (18), the analytical limit of detection for the slowest depleting tissue component [in this case, egg yolk (0.03 ng/g)] was applied as the tolerance for estimating egg withdrawal intervals (WDIs) in this study. In the US, withdrawal times for edible tissues (including muscle, liver, kidney, fat, egg, milk, and honey) are calculated using statistical tolerance limit methods developed by US FDA (“reschem” R package) (17). For FDA's “reschem” R package to calculate an egg discard time, a sufficient number of birds should be used to collect a minimum 10 samples per time point (19). However, the present study only included eight animals which did not lay daily, and only one measurement was performed for each sample at each time point. To satisfy the data format for using the “reschem” package, Crystal Ball (Version 11.1.2.4, Oracle Corporation, Redwood Shores, CA) was used to perform Monte Carlo simulation based on the mean concentration of individual measured egg yolk concentrations and standard deviation of the studied animals at each time point. To accomplish this, all time points with a minimum of 5 samples were selected, and the mean egg yolk concentration and standard deviation were calculated for each time point. Any concentration data below the LOD were excluded in the calculation of WDIs. This data was used to run Monte Carlo simulation to generate additional virtual animals to satisfy the requirement for at least 10 eggs/time point, and this data was entered into the “reschem” package. The WDI was determined when the 99th percentile tolerance limit on the residue concentration was at or below the permitted concentration (i.e., the operational tolerance) with a 95% confidence.

In addition to the US FDA method, WDIs were also calculated using EMA methodology. The EMA has a method for calculating withdrawal times for edible tissues (e.g., liver, kidney, and muscle, WT 1.4 software), and a separate method for milk discard times (WTM 1.4 software). However, no specific method or guidance is currently available for calculating egg withdrawal intervals for the EMA. Since the WTM 1.4 program requires multiple repeated measurements from the same animals, the data from this study was not suitable for the WTM 1.4 as the hens laid irregularly. As a result, this study used the WT 1.4 program to calculate egg yolk WDIs for ivermectin based on EMA methodology. Since the WT 1.4 program only allows for a maximum of 7 timepoint measurements, the final 7 timepoints using the same dataset as for the FDA methodology above were inputted into WT 1.4. Since there is no MRL for ivermectin in eggs in the EU, the LLOQ (0.075 ng/g) for egg yolk was doubled then used in place of an MRL per EMA guidelines (20). In addition, to compare to FDA methodology, the MRL for the EMA method was also set to the analytical LOD (0.03 ng/g) for egg yolk. For EMA methods, the WDI was determined when the 95th percentile tolerance limit of the residue concentration was at or below the permitted concentration (i.e., the LOD) with a 95% confidence. Once the raw WDI estimates were obtained for each methodology, the overall recommended egg discard interval was determined by rounding the WDI estimate up to the nearest 24 h interval.

## 3 Results

### 3.1 Animals

Throughout the entire study period, the hens remained apparently healthy without any visible side effects following ivermectin administration. One hen (bird 7) ceased laying after 47 days. This same hen also had the longest gap between consecutive eggs laid at 27 days. The remainder of the hens laid eggs the entire study period, with an average period between eggs laid of 1.52 days (range 1.16–1.83 days).

### 3.2 Method validation

The LOD, LLOQ, precision, accuracy, intra-assay variation, internal standard recovery, and ivermectin recovery for egg yolk and egg white samples are presented in Table 1. The calibration curve was linear from 0.025 to 50 ng/g with the average *R*^2^ of 0.9975. Selectivity was demonstrated by analysis of blank samples from six individual sources for both egg yolk and egg white and neither showed interfering peaks at the retention times for ivermectin nor the internal standard.

**Table 1:** Sensitivity, precision, and accuracy parameters for the high-performance liquid chromatography analytical method used to measure ivermectin concentrations in egg yolk and egg white following topical ivermectin administration in domestic egg laying hens.

**Parameter**	**Egg yolk**	**Egg white**
LOD (ng/g)	0.03	0.01
LLOQ (ng/g)	0.075	0.025
Precision (%)	1.2	2.3
Accuracy (%)	102.8	101.4
Intra-assay variation (%)	2.3	2.9
Internal standard recovery (%)	76.8	90.6
Ivermectin recovery (%)	81.9	93.1

LOD, Limit of detection; LLOQ, Lower limit of quantification.

### 3.3 Stability testing

Over the 61-day period, ivermectin spiked egg yolk and egg white samples maintained consistent concentrations as indicated through comparison of the RSD values. Average RSD values for ivermectin in the QC refrigerated stability samples were <7% in egg yolks, and <5% in egg whites. The results of the refrigerated sample stability study indicate that there is no significant loss of ivermectin in egg whites or egg yolks for up to 61 days when stored at 4.5°C.

### 3.4 Egg pharmacokinetic analysis

**Figure 1:**
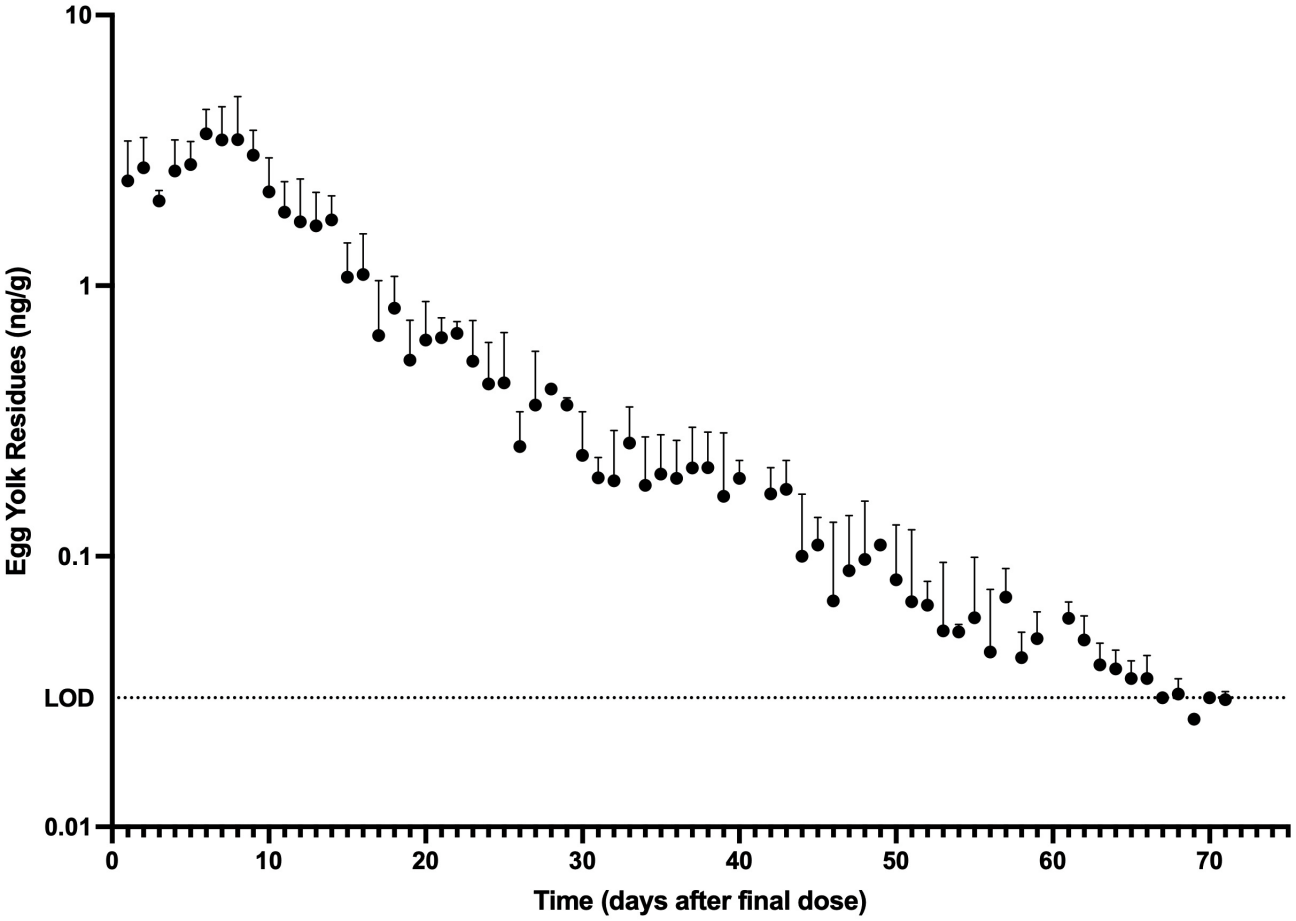
Egg yolk ivermectin residue (mean + standard deviation) vs. time profile obtained after topical administration of ivermectin at 0.4 mg/kg every 7 days for 2 doses to domestic egg laying chickens (*n* = 8). Limit of Detection (LOD): 0.03 ng/g.

**Figure 2:**
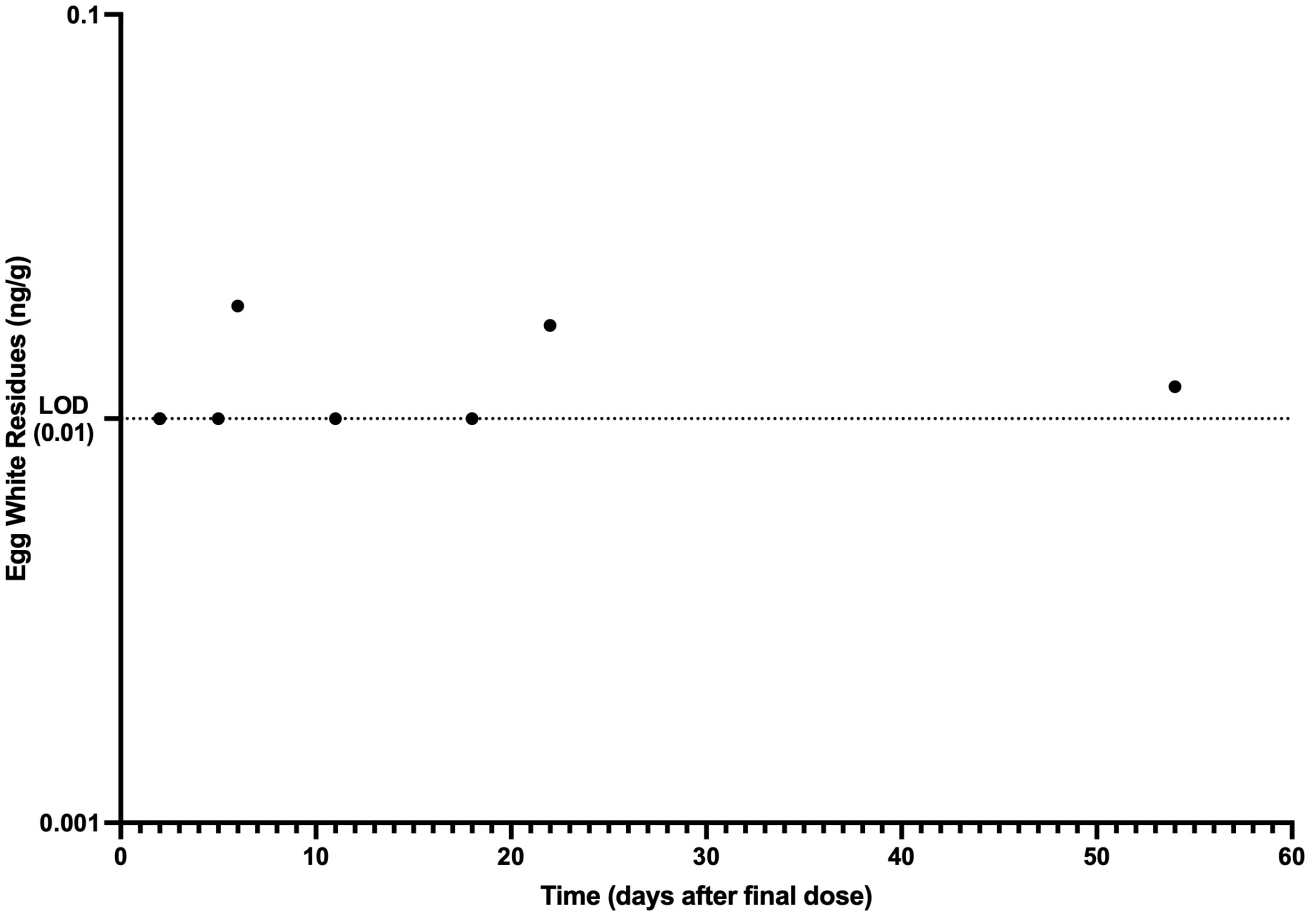
Egg white ivermectin residue vs. time data collected after topical administration of ivermectin at 0.4 mg/kg every 7 days for 2 doses to domestic egg laying chickens (*n* = 8). Limit of Detection (LOD): 0.01 ng/g.

The mean egg yolk and egg white concentration vs. time for laying hens administered injectable ivermectin topically at 0.4 mg/kg every 7 days for two doses are presented in Figures 1, 2, respectively. Overall, ivermectin preferentially distributed to the egg yolk following topical administration in layers. Bird 5 had the fastest depletion of ivermectin from egg yolk, with the last detected ivermectin residues at 39 days following the final administered dose. Bird 3 had the longest depletion of ivermectin from egg yolk with the last detected ivermectin residues at 75 days following the final administered dose. Since only 10 total egg whites had detectable ivermectin residues throughout the entire study period, pharmacokinetic analysis was only performed for the egg yolk data. The associated egg yolk pharmacokinetic parameters are provided in Table 2. Following topical administration, ivermectin residues in egg yolk gradually increased with a geometric mean *C*_max_ of 3.54 ng/g (range 2.5–5.1 ng/g) occurring at a *T*_max_ of 6.6 days (range 6–8 days) after the final dose. Ivermectin egg yolk residues were persistent at low concentrations following topical administration in domestic egg laying chickens, with a geometric mean T1/2 of 228.1 h (range 142.2–297.3 h). The geometric mean AUC0-∞ was 1,513.7 h^*^ng/g (range 1,149.3–2,089.9 h^*^ng/g), and the AUC0-∞ was <6% extrapolated. Given the lack of comparable IV dosing or plasma sampling, limited pharmacokinetic parameters are reported.

**Table 2:** Egg yolk pharmacokinetic parameters estimated by noncompartmental analysis following topical administration of ivermectin at 0.4 mg/kg every 7 days for 2 doses to domestic egg laying chickens (*n* = 8).

**Parameter**	**Egg yolk Geometric mean (range)**
*C*_max(obs)_ (ng/g)	3.54 (2.5–5.1)
*T*_max(obs)_ (h)	159 (144–192)
λ_z_ (1/h)	1.75 (1.3–2.8)
*T*_1/2_ (h)	228.1 (142.2–297.3)
AUC_0−∞_ (h^*^ng/g)	1513.7 (1,149.3–2,089.9)
AUC_0−∞_ Extrapolated (%)	1.0 (0.4–5.5)

C_*max*(*obs*)_, Maximum observed plasma concentration; T_*max*(*obs*)_, Time at which the maximum plasma concentration was observed; λ_*z*_, terminal elimination rate constant; T_1/2_, Terminal elimination half-life; AUC_0−∞_, Area under the plasma concentration-time curve from time 0 to infinity.

### 3.5 Withdrawal interval estimation

Estimated ELDU WDIs using the terminal elimination half-life method, as well as the FDA tolerance and EMA MRL methods are presented in Table 3. The longest estimated egg yolk WDI was 101.7 (rounded to 102) days using the EMA MRL Method and the LOD (0.03 ng/g) as the MRL. The shortest estimated egg yolk WDI was 56.8 days (rounded to 57 days) using the EMA MRL methods with 2 times the LLOQ as the MRL (0.15 ng/g). The FDA tolerance limit method using the LOD as the tolerance (0.03 ng/g) yielded an 80.6 (rounded to 81) day estimated egg yolk WDI, while the terminal elimination half-life method estimated a 95.1 (rounded to 96) day egg yolk WDI. Due to an insufficient number of egg whites with ivermectin concentrations above the LLOQ, a WDI estimation was not possible for this matrix and therefore WDI estimation was only performed for the egg yolk matrix.

**Table 3:** Estimated egg yolk extra-label drug use (ELDU) withdrawal intervals (WDIs) for ivermectin administered topically (0.4 mg/kg every 7 days for 2 doses) to domestic egg laying chickens.

**Method**	**Tolerance/MRL (ppb)**	**Estimated egg yolk WDI (days)**
Terminal elimination half-Life method	Theoretical 99% drug depletion	95.1 (96)
FDA tolerance limit method for LOD (95% confidence interval for 99th percentile)	0.03	80.6 (81)
EMA maximum Residue Limit Method for LOD (95% confidence interval for 95th percentile)	0.03	101.7 (102)
EMA maximum residue limit method for 2x LLOQ (95% confidence interval for 95th percentile)	0.15	56.8 (57)

The terminal elimination half-life method refers to the principle that 99% of drug depletion occurs following 10 half-lives. Therefore, for this method the egg yolk terminal elimination half-life is multiplied by a factor of 10. The EMA method refers to the computerized version of the method (WT 1.4 software) used to calculate withdrawal periods for tissues developed by the Committee for Veterinary Medicinal Products (CVMP) of the European Medicines Agency (EMA). The FDA method refers to the tolerance limit method (coded in the “reschem” R package) used to calculate tissue withdrawal times developed by the US Food and Drug Administration (FDA). Both FDA and EMA methods outputs the exact time that drug concentration is below the tolerance or maximum residue limit, and this time is rounded to the next 24-hour interval (shown in parenthesis).

## 4 Discussion

Following administration of the injectable formulation of ivermectin at 0.4 mg/kg topically every 7 days for 2 doses to domestic egg laying chickens, ivermectin was systemically absorbed and did distribute to the eggs. Residues preferentially distributed to the egg yolk and persisted at low concentrations for up to 71 days after the final dose. This is the first study to evaluate egg residues of ivermectin following topical administration, and to demonstrate that the commercially available injectable formulation of ivermectin is systemically absorbed following topical administration in poultry. There were no adverse clinical effects noted following topical administration of the injectable product to chickens in this study. Following topical administration, elimination of ivermectin from egg yolk was very slow, with persistent residues at low concentrations resulting in a terminal elimination half-life of 9.5 days. Despite being administered at a higher dose (0.4 mg/kg), topical administration of injectable ivermectin in this study resulted in much lower maximum ivermectin residues in eggs compared to a previous study using single dose (0.2 mg/kg) IV (*C*_max_ ~15.5 ng/g) and single dose (0.2 mg/kg) SC (*C*_max_ ~23.2 ng/g) (15). The maximum ivermectin residues in eggs following topical administration for the injectable formulation in this study at 0.4 mg/kg were slightly higher than single dose oral administration of the injectable formulation (0.2 mg/kg) in fed chickens (*C*_max_ ~1.8 ng/g) in a previous study (15). However, it should be noted that a higher dose was used in this study compared to previous, and the egg yolks and whites were analyzed separately in this study, while the previous oral study homogenized egg yolks and whites prior to analysis—likely leading to dilution of ivermectin concentrations (15).

Similar to other studies using other routes of administration, ivermectin demonstrated preferential distribution to the egg yolk following topical administration with minimal detectable concentrations of ivermectin in egg white (9, 16). In general, drug characteristics resulting in distribution into the egg yolk include increasing lipid solubility, increasing binding to yolk lipoproteins, the drug's molecular weight, and chemical structure (21, 22). Ivermectin is known for its extensive distribution, lipophilicity, and high plasma protein binding characteristics, leading to accumulation in fat and other tissues, such as egg yolks. Ivermectin residues were much more persistent in egg yolks following topical administration in egg laying chickens compared to previous studies for other routes of administration, with residues detectable for 8 days following single dose oral and intravenous administration (15), 15 days following single dose subcutaneous administration (15), and 20 days following multi-day administration as a medicated water (9). While the reason why ivermectin residues in eggs are more persistent following topical administration is not fully understood, it can be postulated that a combination of altered pharmacokinetics following topical administration and the much lower LOD in this study (0.03 ppb) compared to previous studies (0.06 and 0.2 ppb, respectively) may play a role. In general, drug disappearance from egg yolks occurs ~10 days after disappearance of drugs from the plasma, although this is dependent on the magnitude of plasma drug concentrations, the stage of yolk development at drug exposure, and the sensitivity of the analytical method (21). Since this study did not evaluate plasma kinetics in hens following topical administration of ivermectin, it is unknown if the egg yolk depletion profile mirrors the plasma kinetics for ivermectin *via* this route. However, it is likely that ivermectin behaves similarly to other highly lipophilic drugs, such as fipronil, and that ivermectin residues distribute during phase 2 of yolk development when lipoproteins from the liver begin to accumulate in the yolk (21, 23). Since ivermectin accumulates in fat, redistribution of ivermectin from fat likely leads to continued accumulation of yolk residues until the egg is laid 6 weeks later (21).

The persistence of ivermectin residues in egg yolk following topical administration led to prolonged estimated WDIs for all approaches. Since this is a pilot study, the study design does not meet the requirements of EMA or FDA for the drug approval process (19, 20). Part of the approach for WDI estimation using this pilot study data was to use a Monte Carlo simulation method to generate the sample size of 10 eggs/timepoint that is required for FDA methods through simulated data using the mean concentration of individual measured egg yolk concentrations and standard deviation of the studied animals at each time point. While this approach does not replace adequate sample size or FDA/EMA requirements, it does provide framework for estimating a conservative withdrawal interval following extra-label drug use of ivermectin following topical administration in chickens.

The longest estimated egg WDI was 102 days using the EMA MRL method with the MRL set at the LOD, while the shortest estimated egg WDI was 57 days using the EMA MRL method with the MRL set at twice the LLOQ of the present study. Since the same regulatory method generated the longest and shortest WDI estimates, this highlights the effect of MRL/tolerance on WDI estimates following ELDU in food producing species. The FDA tolerance limit method, with the LOD as the set tolerance, resulted in a shorter estimated WDI (81 days) than the EMA MRL method with the MRL set at the LOD (102 days). This first seems counterintuitive because the EMA MRL method is based on the 95% confidence interval for the 95th percentile of the population, while the FDA tolerance limit method is based on the 95% confidence interval for the 99th percentile of the population. Therefore, the EMA method should result in a less conservative WDI estimate because it minimizes the weight of extreme outliers in comparison to the FDA method. However, the differences in this case are likely due to the sampling window that each of the regulatory programs allow. The EMA WT 1.4 program uses the final 7 timepoints of the elimination phase, while the FDA “reschem” R package allows for entry of all elimination phase timepoints. Therefore, because ivermectin egg yolk residues were persistent at low concentrations hovering around the LOD in this study, the EMA WT 1.4 program likely resulted in overestimation of the terminal portion of the elimination phase in comparison to the FDA “reschem” R package. The terminal elimination half-life method resulted in an egg WDI estimation of 96 days, falling roughly between the FDA tolerance limit method (LOD) and the EMA MRL method (LOD).

For all calculation methods, even the shortest estimated egg withdrawal interval chickens treated with topical ivermectin administration is very long, precluding any functional use in layers for commercial production. It should be noted that the hens in this pilot study were older hens with inconsistent egg production, with the average period between eggs laid to be 1.52 days (range 1.16–1.83 days). While these hens were chosen to be reflective of a typical backyard flock, ivermectin depletion following topical administration in younger hens that consistently lay eggs on a daily basis may be more rapid, resulting in a shorter estimated WDI. Since there is no MRL or tolerance for ivermectin in eggs in the EU or US, the targeted MRL/tolerance defaults to regulatory guidelines, which is twice the LLOQ of the analytical method in the EU per EMA guidelines and is the LOD of the analytical method in the US (18, 20). These regulatory guidelines are based on the concept that, in the absence of an approved product for that species/matrix, the only way to ensure human food safety is for there to be no detectable residues. Therefore, the safe level in a food product is driven by the analytical method rather than an MRL/tolerance derived from a human food safety risk assessment.

As analytical methods become more sensitive over time, the WDI required for drugs to deplete below the LOD or LLOQ following ELDU have also significantly increased. In this study, the analytical LOD for ivermectin in egg yolk was 0.03 ng/g (0.03 ppb). Since ivermectin consists of roughly 80% 22,23-dihydroavermectin B_1a_ (H2B1_a_) and 20% 22,23-dihydroavermectin B_1b_, the marker residue to monitor for total ivermectin residues for both the US and EU is H2B1_a_ (24). In the US, the tolerance for ivermectin is based on the acceptable daily intake (ADI) of 5 ug/kg/day total ivermectin residues (25). This ADI results in a US tolerance for H2B1_a_ of 1,600 ppb for the marker tissue (liver) in cattle, 30 ppb in sheep, and 20 ppb in swine (25). In the EU, the ADI for ivermectin is considered to be 10 ug/kg/day total ivermectin residues. This results in an EMA MRL for H2B1_a_ of 100 ppb for the liver and fat, and 30 ppb for the muscle and kidney of all mammalian food producing species (26). Based on these regulatory approaches for deriving a tolerance/MRL, a non-regulatory alternative approach to provide a provisionally acceptable residue (PAR) limit has been described for extralabel use of compounds in food producing animals in the US for compounds in which an ADI has been established (24). Based on these calculations and the established ADI in the US (5 ug/kg/day), the partitioning factor of the ADI for eggs (20%), the FDA food consumption value for eggs (0.1 kg/day), and the established human bodyweight used in FDA calculations (60 kg), a PAR for ivermectin in eggs could be considered to be 600 ppb for total ivermectin residues, which is much higher than any residues detected in this study (24). However, since this study did not quantify H2B1_a_ in egg yolks, this PAR is not reflective of the tolerance that may be assigned for the marker residue H2B1_a._ Further studies to elucidate the presence and magnitude of H2B1_a_ residues in the egg yolk following topical administration to chickens are warranted.

There are several limitations to this study. Since the design of this study does not specifically meet the FDA guidance for industry guidelines or EMA regulatory requirements for sponsors seeking drug approval, the estimated egg withdrawal intervals from this study should only serve as withdrawal estimates following ELDU. Additional studies that meet the EMA or FDA study requirements are needed to make a more conclusive evidence-based recommendation for large flocks of birds. Additionally, due to the irregular egg production and small sample size available, additional simulated samples were used to estimate the WDI. While these sample points are based on Monte Carlo simulation of this population and are modeled off a worst-case scenario (older hens, irregular egg production), the extrapolation of these recommendations to more diverse populations/production systems should be done with caution. Further studies exploring the plasma and tissue pharmacokinetics of ivermectin following topical administration are needed. Additionally, further studies involving larger populations of younger hens with regular egg production are needed.

## 5 Conclusion

Ivermectin is systemically absorbed following topical administration of the injectable formulation in domestic egg laying chickens, resulting in prolonged egg residues. Ivermectin is preferentially distributed to the egg yolk over the egg white following topical administration of the injectable formulation in egg laying chickens. Following topical administration of injectable ivermectin formulation to egg laying hens (0.4 mg/kg every 7 days for 2 doses), an egg withdrawal interval of 81 days was estimated based on the FDA tolerance limit method targeting the analytical limit of detection (0.03 ng/g). Overall, this study improves the understanding of the residue depletion kinetics of ivermectin in eggs after topical administration. Additionally, the results provide insight into how the estimated ivermectin egg WDIs using regulatory methods differ based on the maximum residue limit/tolerance applied and portion of the terminal elimination phase sampled.

## Data Availability

The raw data supporting the conclusions of this article will be made available by the authors, without undue reservation.
